# Metformin attenuates silica-induced pulmonary fibrosis via AMPK signaling

**DOI:** 10.1186/s12967-021-03036-5

**Published:** 2021-08-16

**Authors:** Demin Cheng, Qi Xu, Yue Wang, Guanru Li, Wenqing Sun, Dongyu Ma, Siyun Zhou, Yi Liu, Lei Han, Chunhui Ni

**Affiliations:** 1 Department of Occupational Medical and Environmental Health, Key Laboratory of Modern Toxicology of Ministry of Education, Center for Global Health, School of Public Health, Nanjing Medical University, Nanjing, 211166 China; 2 Institute of Occupational Disease Prevention, Jiangsu Provincial Center for Disease Control and Prevention, Nanjing, 210028 China

**Keywords:** Occupational pulmonary fibrosis, Metformin, Epithelial-mesenchymal transition, Fibroblast-myofibroblast transition

## Abstract

**Background:**

Silicosis is one of the most common occupational pulmonary fibrosis caused by respirable silica-based particle exposure, with no ideal drugs at present. Metformin, a commonly used biguanide antidiabetic agent, could activate AMP-activated protein kinase (AMPK) to exert its pharmacological action. Therefore, we sought to investigate the role of metformin in silica-induced lung fibrosis.

**Methods:**

The anti-fibrotic role of metformin was assessed in 50 mg/kg silica-induced lung fibrosis model. Silicon dioxide (SiO_2_)-stimulated lung epithelial cells/macrophages and transforming growth factor-beta 1 (TGF-β1)-induced differentiated lung fibroblasts were used for in vitro models.

**Results:**

At the concentration of 300 mg/kg in the mouse model, metformin significantly reduced lung inflammation and fibrosis in SiO_2_-instilled mice at the early and late fibrotic stages. Besides, metformin (range 2–10 mM) reversed SiO_2_-induced cell toxicity, oxidative stress, and epithelial-mesenchymal transition process in epithelial cells (A549 and HBE), inhibited inflammation response in macrophages (THP-1), and alleviated TGF-β1-stimulated fibroblast activation in lung fibroblasts (MRC-5) via an AMPK-dependent pathway.

**Conclusions:**

In this study, we identified that metformin might be a potential drug for silicosis treatment.

**Supplementary Information:**

The online version contains supplementary material available at 10.1186/s12967-021-03036-5.

## Introduction

Silicosis is one of the most common occupational pneumoconiosis directly caused by exposure to respirable silica-based particles, which leads to an inflammatory cascade, progressive lung fibrosis, and then devastated respiratory failure [1]. Persistent inflammation, epithelial-mesenchymal transition (EMT), fibroblast proliferation and activation, and extracellular matrix (ECM) excessive deposition contribute to the development of silicosis [2]. Although efforts to improve the work environment have been made for years, the incidence and prevalence of silicosis worldwide have been rising, especially in developing countries like China [3, 4]. However, the underlying pathogenesis of silicosis is still not elucidated and none of the effective therapies can halt disease progression or reverse established lung fibrosis. There is yet an urgent need for developing efficient methods.

Over the past decades, mounting evidence indicates that several pathogenic mechanisms are known to be involved in the progression of silicosis [5]. Multiple studies have identified that a variety of cells include epithelial cells, macrophages, and pulmonary fibroblasts, are participating in the process of silica-induced pulmonary fibrosis [6, 7]. EMT entails the phenotypic changes and molecular reprogramming which characterize the conversions of epithelial cells to mesenchymal cells [8]. The current notion suggested that epithelial cells participated in the development of myofibroblasts via the EMT process [9]. EMT can activate transcription factors, secrete pro-fibrotic cell surface proteins and cytokines, and increase ECM accumulation [10–12]. Macrophage is one of the most important effector cells in silicosis, which is continuously activated via silica stimulation, secreting multiple inflammatory factors and exacerbating silicosis [13]. Macrophages play a crucial role in the progression of pulmonary fibrosis by triggering the inflammation cascade response and differentiating into a profibrotic phenotype [14, 15]. Silica damages the epithelial cell and also activates the macrophage to cause an inflammatory microenvironment in the lung, which promotes adaptive immunity, fibroblast proliferation and migration, extracellular matrix secretion and collagen deposition, eventually leading to abnormal lung tissue remodeling and obstructive air exchange, even death [16, 17]. The fibroblast to myofibroblast transition is the hallmark of fibrotic diseases, leading to excessive synthesis and deposition of ECM like Collagen, Fibronectin and Elastin [18, 19]. Thus, suppression of the EMT process, inflammatory cascade response and fibroblast activation represent a visible approach for the amelioration of pulmonary fibrosis.

Metformin’s history is linked to Galega officinalis, traditional herbal medicine in the temperate climes of Western Asia and Southern Europe, which, was used to lower blood glucose clinically in the 1920s [20]. Numerous studies demonstrated that metformin stimulates AMP-activated protein kinase (AMPK), a key regulator of energy metabolism and balance [21, 22]. Interestingly, recent studies showed that metformin could also exert effects independent of the AMPK pathway, for example, by inhibiting mitochondrial glycerophosphate dehydrogenase and mitochondrial respiration [23]. In several experimental animal models, apart from type 2 diabetes mellitus (T2DM), metformin was found to have other potential effects. Metformin has been acted in other pathologies like cancer, non-alcoholic fatty liver disease, asthma, heart failure, obesity, fibrotic diseases, etc., with varying degrees of effect [24–28]. It is interesting to note that metformin exerts protective effects in various lung diseases by inhibiting inflammation, attenuating oxidative stress, and altering fibroblast-to-myofibroblast transition [29, 30]. Moreover, it has been reported that metformin could reverse established pulmonary fibrosis in the bleomycin-induced mouse lung fibrosis model [31] and low prevalence of pneumoconiosis in diabetic coal-miners [32]. Another recent report investigated that metformin could downregulate the pro-fibrotic cytokine [like transforming growth factor-beta 1(TGF-β1)] in the kidney by increasing the level of butyrate in the systemic circulation [33]. We concluded the metformin/AMPK downstream signalings in fibrotic diseases in Additional file 1: Figure S1. Given this, it appears that metformin can attenuate the key factors leading to fibrosis. Hence, we considered that metformin might be useful in silica-induced pulmonary fibrosis both in vivo and in vitro.

In this study, we evaluated the anti-fibrotic effects of metformin in the silica-induced lung fibrosis model at different stages to illustrate the underlying mechanisms in different cell types, including pulmonary epithelial cells, macrophages, and fibroblasts. Functionally, we demonstrated the evidence that metformin did ameliorate the cell toxicity, inflammation, epithelial-mesenchymal transition, and oxidative stress caused by silica and fibroblast activation induced by TGF-β1. Collectively, metformin may have the therapeutic potential for ameliorating established lung fibrosis.

## Material and methods

### Metformin treatment mouse model of the late fibrotic stage

A total of 24 mice were randomly divided into four groups (n = 6 each group, using Excel 2010 software random number formula): a saline group, a silica group, a 100 mg/kg of metformin plus silica group, and a 300 mg/kg of metformin plus silica group. All the mice in the silica groups were received a single intratracheal instillation with 50 mg/kg of silica particles (size distribution: 99% between 0.5 and 10 μm, 80% between 1 and 5 μm, average particle diameter 1.7 μm, obtained from Sigma-Aldrich, St. Louis, MO, USA) dissolved in 0.05 ml sterile saline. After silica treatment for 28 days, the mice of metformin treatment groups were given a corresponding dose of metformin (started from the 28th day, Beyotime Institute of Biotechnology, Shanghai, China) via intragastrical administration daily for 2 weeks. Then the mice were sacrificed after installation, and the lung tissues were isolated and stored at – 80 °C for further analysis.

### Metformin treatment mouse model of the early fibrotic stage

Another 24 mice were subdivided into four groups (n = 6 each group): saline, silica, silica plus saline (used as negative control of silica plus metformin), and silica plus metformin (300 mg/kg, intragastrical administration for 28 days). The method of silica treatment was the same as mentioned above. The silica plus saline and silica plus metformin group were treated via intragastrical administration saline or metformin daily after silica treatment the next day. The mice were sacrificed after the installation treatment for 4 weeks, and the lung tissues were harvested and stored as previously described.

### Histopathology

The mouse lung tissues were harvested, fixed with formalin solution overnight and embedded in paraffin. Then the tissues sections (5 μm) were subsequently stained with hematoxylin and eosin (H&E) to assess fibrosis. To evaluate the pathological changes of the lung tissues, a scoring system was used as previously described [34], lesion severity: 0 = zero/nothing, 1 = marginal, 2 = slight, 3 = moderate, 4 = severe, 5 = very severe; lesion distribution: 0 = absent, 1 = rare/occasional (10% of the lung area), 2 = spares/limited (10–25% of the lung area), 3 = moderate (25–50% of the lung area), 4 = extensive/widespread (50–75% of the lung area), 5 = very extensive/predominant (over 75% of the lung area).

### Hydroxyproline content assay

The amounts of collagen in the lung tissues were determined by the hydroxyproline content assay (A030-2, Jiancheng Bioengineering Institute, Nanjing, China). According to the manufacturer’s protocol, the amount of collagen was determined by the spectrophotometer at 550 nm and expressed as micrograms per mg of lung tissues.

### Cell culture and treatment

The lung epithelial cells (A549 and HBE), lung fibroblast (MRC-5), and the human monocytic cell (THP-1) were commercially obtained from the American Type Culture Collection (ATCC, Manassas, VA, USA). A549 and THP-1 cells were maintained in RPMI Medium 1640 basic (1640, Life Technologies/Gibco, Grand Island, NY, USA), the HBE cells were maintained in Dulbecco’s modified Eagle’s medium (DMEM, 1640, Life Technologies/Gibco, Grand Island, NY, USA), and the MRC-5 cells were maintained in Minimum Essential Medium (MEM, Life Technologies/Gibco, Grand Island, NY, USA). All of the culture media were containing 10% fetal bovine serum (BISH1475, Biological Industries) and antibiotics (penicillin and streptomycin, Life Technologies/Gibco, Gaithersburg, MD). Cells were cultured at 37 °C in a 5% CO_2_ atmosphere.

PMA (Sigma–Aldrich) was used to treat THP-1 cells into macrophages. For all the experiments analysis, epithelial cells and THP-1 were treated with 200 or 150 μg/ml Silicon dioxide (SiO_2_) (Sigma-Aldrich, St. Louis, MO, USA) together with various concentrations (2, 5, 10 mM) of metformin (Beyotime Institute of Biotechnology, Shanghai, China); MRC-5 cells were treated with 5 ng/ml TGF-β1 (Sigma-Aldrich) together with metformin.

### Gene silencing and drug treatment

AMPK siRNA was synthesized by Generay Biotech (Shanghai, China). Cells were transiently transfected using riboFECT CP Regent (RiboBio, Guangzhou, China) according to the manufacturer’s instructions. The sequence of si-AMPK: sense: 5′-GUUGCCUACCAUCUCAUAAUATT-3′; antisense: 5′-UAUUAUGAGAUGGUAGGCAACTT-3′; AMPK inhibitor Compound C was phased from MedChemExpress.

### Cell viability assay

Cell viability was detected with a Cell Counting Kit-8 assay (CCK8, Beyotime Institute of Biotechnology, Shanghai, China) according to the manufacturer’s instructions. The cells were plated in a 96-well plate, followed by exposure to different concentrations (0, 50, 100, 150 and 200 μg/ml) of silica and (2 mM, 5 mM and 10 mM) of metformin for the indicated times. Then 10 μl CCK8 reagents were diluted in each well for 1 h at 37 °C in 5% CO_2_, and the 96-well plate was measured at 450 nm carried out with a microplate reader (TECAN Infinite M200, Mannedorf, Switzerland). The percent viability of the cells was calculated by the formula: cells viability (%) = (A_450nm_ − A_650nm_) _treated_/(A_450nm_ − A_650nm_) _control_ × 100%.

### Immunostaining assay

After the indicated treatment, A549 or HBE cells were washed fixed with 4% methanol for 30 min, then stained with anti-Vimentin or anti-E-cadherin antibody (Abcam, ab92547, 1:1000; Cell Signaling Technology, 24E10, 1:1000) at 4 °C overnight and incubated with Cy3-conjugated or FITC-conjugated goat anti-rabbit antibody (1:200, Beyotime Institute of Biotechnology, Shanghai, China) for 1 h. The DAPI was used to stain the nucleus in cells for 10 min. All the images were acquired with the fluorescence microscope (Olympus, Tokyo, Japan).

### Western blot and antibodies

For western blot assay, all cells were washed twice times with PBS and then used with RIPA lysis buffer and phenylmethylsulfonyl fluoride for extraction of total proteins (Beyotime Institute of Biotechnology, Shanghai, China; PMSF, Sigma-Aldrich, St. Louis, MO, USA). The total protein of the mouse lung tissues was extracted with T-PER Tissue Protein Extraction Reagent (Thermo Scientific). Protein concentrations were measured by BCA Protein Assay (Beyotime Institute of Biotechnology, Shanghai, China). A total of 80 μg of protein extracts were separated via SDS-PAGE and transferred onto polyvinylidene difluoride (PVDF) membranes (ISEQ00010, 0.2 μm, Immobilon). Then the membranes were incubated overnight at 4 °C with appropriate primary antibodies and appropriate secondary antibodies. Protein bands were visualized using the ChemiDocXRS + imaging system (Bio-Rad Laboratories, Inc). Densitometry analyses were performed using Image J software.

Primary antibodies are as follows: anti-Fibronectin (Abcam, ab45688, 1:1000); anti-Collagen I (Abcam, ab34710, 1:1000; ABclonal, A1352, 1:1000); anti-E-cadherin (Cell Signaling, 24E10, 1:1000); anti-Vimentin (Abcam, ab92547, 1:1000); anti-αSMA (Abcam, ab32575, 1:1000); p-STAT3 (Abcam, ab76315, 1:1000); STAT3 (Cell Signaling, 4904, 1:2000); TGF-β1 (Abcam, ab215715, 1:1000); p-AMPK (Cell Signaling, 4188, 1:2000); AMPK (Cell Signaling, 5831, 1:1000) and anti-GAPDH (Cell Signaling, 13E5, 1:1000).

### Wound-healing assay

Cells were seeded in 6-well plates and cultured until the cells reached 70–80% confluence. Wounds were scratching with a sterile 10 μl pipette tip across the monolayered cells to create a straight linear scratch, and a wound gap was performed by a microscope. After indicated treatment, the widths of the wound were followed by the previously described procedure. The wound gap was quantitatively evaluated with Image J software.

### Quantitative RT-PCR (qRT-PCR)

Total RNA from collected tissues or cells was extracted using the Trizol method as previously described [7]. RNA quality and concentration were measured by Nanodrop 2000 spectrophotometer (ND-100, Thermo, Waltham, MA). All mRNA was detected using AceQ qPCR SYBR Green Master Mix (Vazyme Biotech Co, Nanjing, China) in the ABI 7900HT Real-Time PCR system (Applied Biosystems). Fold changes in the expression levels were calculated using the 2^−∆∆Ct^ method and normalized using GAPDH as the endogenous control.

### Reactive oxygen species assay

The intracellular reactive oxygen species (ROS) level was measured by ROS Assay Kit (Beyotime Institute of Biotechnology, Shanghai, China), using an oxidation-sensitive fluorescent probe DCFH-DA according to the manufacturer’s instructions. The intensity of fluorescent staining was quantified with Image J software.

### Lipid peroxidation assay

The lipid peroxidation product malondialdehyde (MDA) concentration in cell lysates was measured using a Lipid Peroxidation MDA Assay Kit (Beyotime Institute of Biotechnology, Shanghai, China) according to the manufacturer’s instructions.

### Glutathione assay

The relative glutathione (GSH) concentration in cell lysates was measured using a Total Glutathione Assay Kit (Beyotime Institute of Biotechnology, Shanghai, China) according to the manufacturer’s instructions.

### SOD, GSH-Px and CAT assay

The expression levels of superoxide dismutase (SOD), glutathione peroxidase (GSH-Px) and catalase (CAT) in cell supernatant were measured using SOD, GSH-Px and CAT commercial kits (Nanjing Jiancheng Bioengineer Institute, China) according to the manufacturer’s instructions.

### JC-1 staining

JC-1 fluorescent probe was employed to measure the mitochondrial depolarization in cells. The mitochondrial membrane potential of cells using a mitochondrial membrane potential assay kit with JC-1 (Beyotime Institute of Biotechnology, Shanghai, China) according to the manufacturer’s instructions. Cell treated with CCCP (mainly cause loss of mitochondrial membrane potential) was used as a positive control.

### LDH release assay

The cell of cytotoxicity was measured by the lactate dehydrogenase (LDH) activity in the supernatant using an LDH Cytotoxicity Assay Kit (Beyotime Institute of Biotechnology, Shanghai, China) according to the manufacturer’s instructions.

### Statistical analysis

All experiments were performed in triplicates. Statistical analysis was performed using Student’s t-test (between two groups) or one-way analyses of variance followed by Tukey’s multiple comparisons test (more than two groups). Data were expressed as mean ± SD. *P* < 0.05 was considered statistically significant.

## Results

### Metformin attenuates SiO_2_-induced lung fibrosis in vivo

**Fig. 1:**
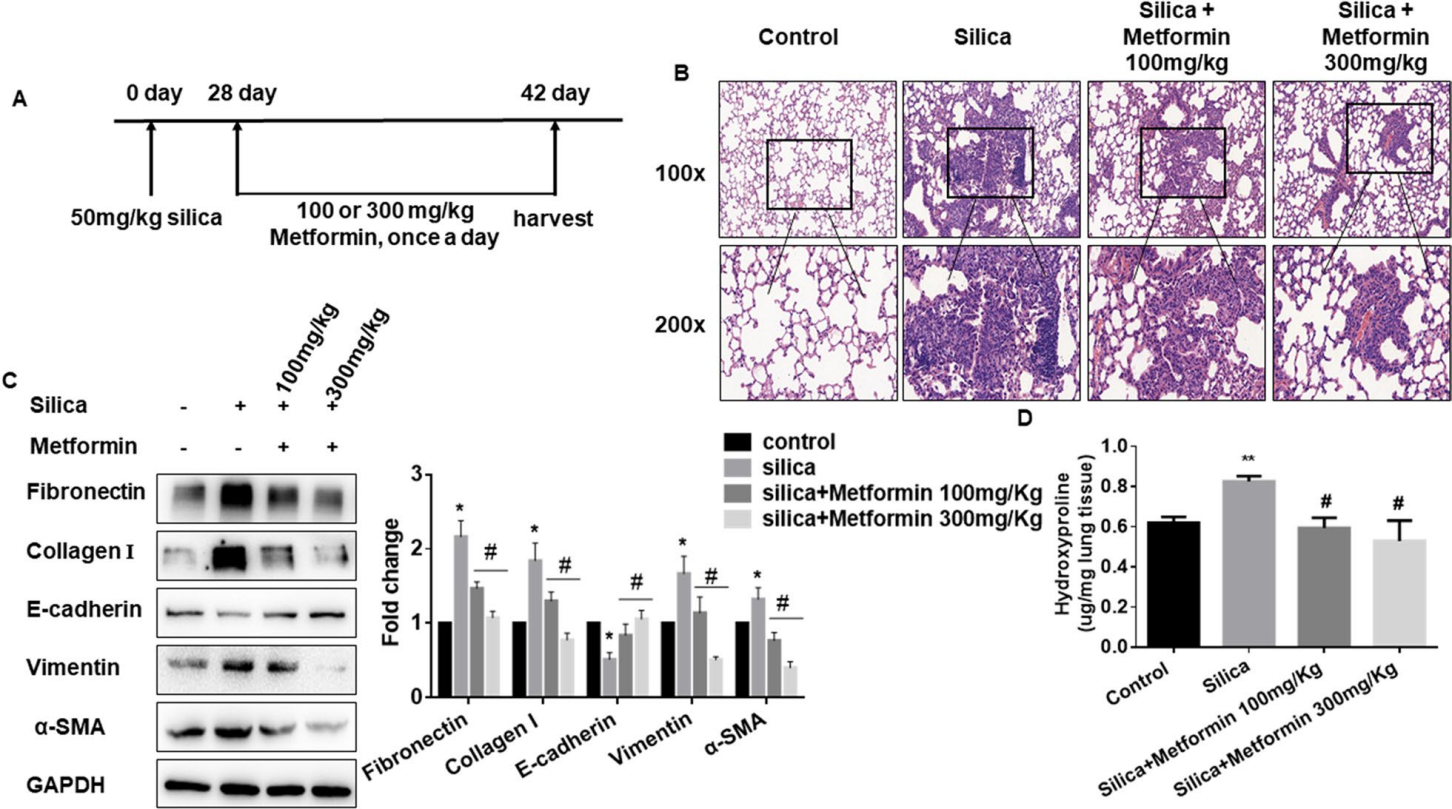
Metformin attenuates SiO_2_-induced lung fibrosis in vivo in the late fibrotic stage. **A** Schematic diagram of the metformin treatment mouse model. **B** H&E staining reflected that the histological changes of lung tissues of different groups. **C** The protein levels of Fibronectin, Collagen I, E-cadherin, vimentin, and α-SMA in each group, with *p < 0.05 vs. control and ^#^*p* < 0.05 vs. the silica group. **D** Hydroxyproline content of the lung tissues in each group was detected to measure collagen deposition, with **p < 0.01 vs. control and ^#^*p* < 0.05 vs. the silica group

To elucidate whether metformin has a potential role in silica-induced pulmonary fibrosis, we established an intervention model by giving 100 or 300 mg/kg metformin to mice after silica-instillation for 28 days. We continued the treatment for two weeks and observed a 100% survival rate and no obvious body weight difference in all groups (Fig. 1A and Additional file 2: Figure S2A). Representative H&E staining showed fibrotic remodeling characteristics by accumulating typical fibrotic nodules and abnormal alveolar structure in the silica group. Notably, metformin (100 or 300 mg/kg) intervention gradually attenuated severe histological changes (Fig. 1B). Moreover, metformin also reduced the degree of severity and distribution of lung lesions in silica-treated mice (Table 1). Consistently, the elevated expression of fibrotic markers (including α-SMA, Collagen I, Fibronectin, etc.) was significantly decreased in a metformin dose-dependent manner (Fig. 1C). The qRT-PCR results indicated that metformin also reduced the mRNA level of inflammation marker [tumor necrosis factor-alpha (TNF-α) and interleukin-1 beta (IL-1β)] in the lung tissues compared with the silica group (Additional file 2: Figure S2B and C). The collagen deposition levels were also observed in metformin intervention groups through hydroxyproline assay and indicated that metformin inhibited silica-induced the content of collagen deposition (Fig. 1D).

**Table 1:** Effect of Metformin administration on lung histopathology in the late stage

Groups	Lesion severity grade						Average severity grade	Lesion distribution grade						Average distribution grade
	0	1	2	3	4	5		0	1	2	3	4	5	
Saline	6						–	6						–
Silica				1	2	3	4.33 ± 0.33			1	1	3	1	3.67 ± 0.42
Silica + 100 mg/kg metformin		2			4		3.67 ± 0.84		2	4				1.67 ± 0.21^a^
Silica + 300 mg/kg metformin	1	3	1			1	1.67 ± 0.71^a^	1	4	1				1.00 ± 0.26^a^

Values represent the means ± SD of 6 for each group

^a^*p* < 0.05 vs. silica group (independent-sample *t*-test)

**Fig. 2:**
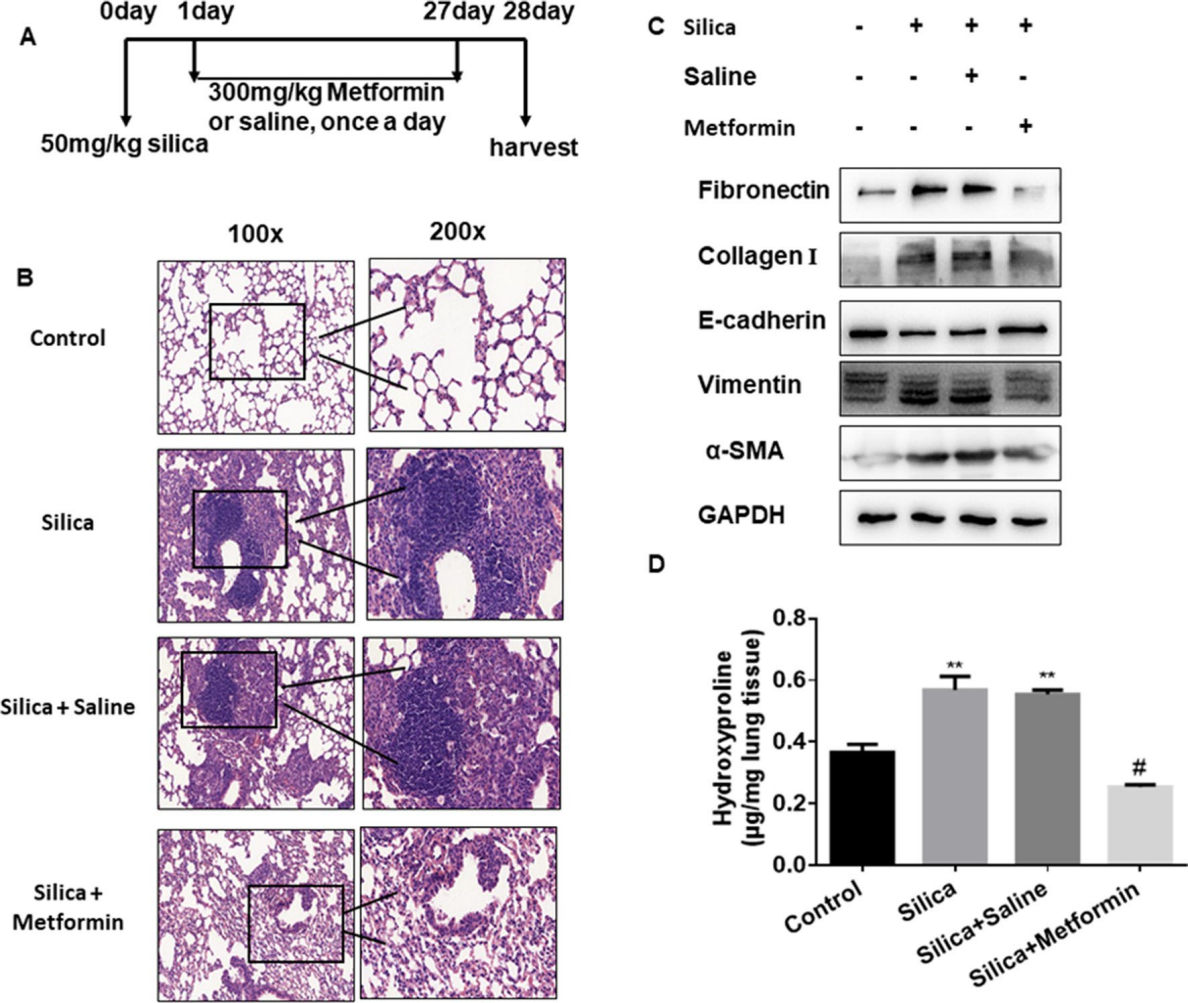
Metformin prevents SiO_2_-induced lung fibrosis in vivo in the early fibrotic stage. **A** Schematic diagram of metformin intervention in the silica-induced pulmonary fibrosis mouse model. **B** The histology of lung tissues was detected by H&E staining in different groups. **C** The protein levels of Fibronectin etc. in lung tissues via western blotting assay. **D** The collagen deposition of different groups was performed by hydroxyproline content assay, with **p < 0.01 vs. control and ^#^*p* < 0.05 vs. the silica plus saline group

Next, we investigated the role of metformin (300 mg/kg was chosen) in the early fibrotic stage. The early-stage therapeutic effect of metformin was applied in mice following silica administration the next day. Mice were treated with metformin or saline from day 2 to day 28, then collected lung tissues for the following analysis on day 28 (Fig. 2A). We have established the metformin administration at different times (7, 14, 28 days) model in the early stage of fibrosis. However, we did not observe obvious fibrotic changes on the day of 7 and 14 days of metformin administration (Additional file 3: Figure S3A). And, administration of metformin in the early stage (at 28 days) significantly alleviated silica-induced pulmonary injury and fibrosis as illustrated by the H&E staining of lung histopathological sections as well as the histologic scores of severity/distribution of the lung lesions (Fig. 2B and Table 2). Similarly, mice pretreatment with metformin displayed a significant decrease of fibrotic markers (α-SMA, Collagen I, Fibronectin) and epithelial-mesenchymal transition marker, vimentin (Fig. 2C and Additional file 3: Figure S3B), coupled with lower content of hydroxyproline in the lung tissues (Fig. 2D). Of note, much lower levels of inflammation marker, TNF-α and IL-1β, were detected in the metformin intervention group compared to the silica group (Additional file 3: Figure S3C and D). Collectively, our results supported that metformin could be an effective therapy against silica-induced lung fibrosis in different stages of fibrotic.

**Table 2:** Effect of Metformin administration on lung histopathology in the early stage

Groups	Lesion severity grade						Average severity grade	Lesion distribution grade						Average distribution grade
	0	1	2	3	4	5		0	1	2	3	4	5	
Saline	6						–	6						–
Silica				2	3	1	3.83 ± 0.307			1	3	1	1	3.33 ± 0.42
Silica + saline				1	4	1	4.00 ± 0.25			3	2	1		2.67 ± 0.33
Silica + metformin	1	2	3				1.33 ± 0.33^a^	2	3	1				0.83 ± 0.32^a^

Values represent the means ± SD of 6 for each group

^a^*p* < 0.05 vs. Silica + Saline group (independent-sample *t*-test)

### Metformin attenuates SiO_2_-induced cell cytotoxicity in vitro

**Fig. 3:**
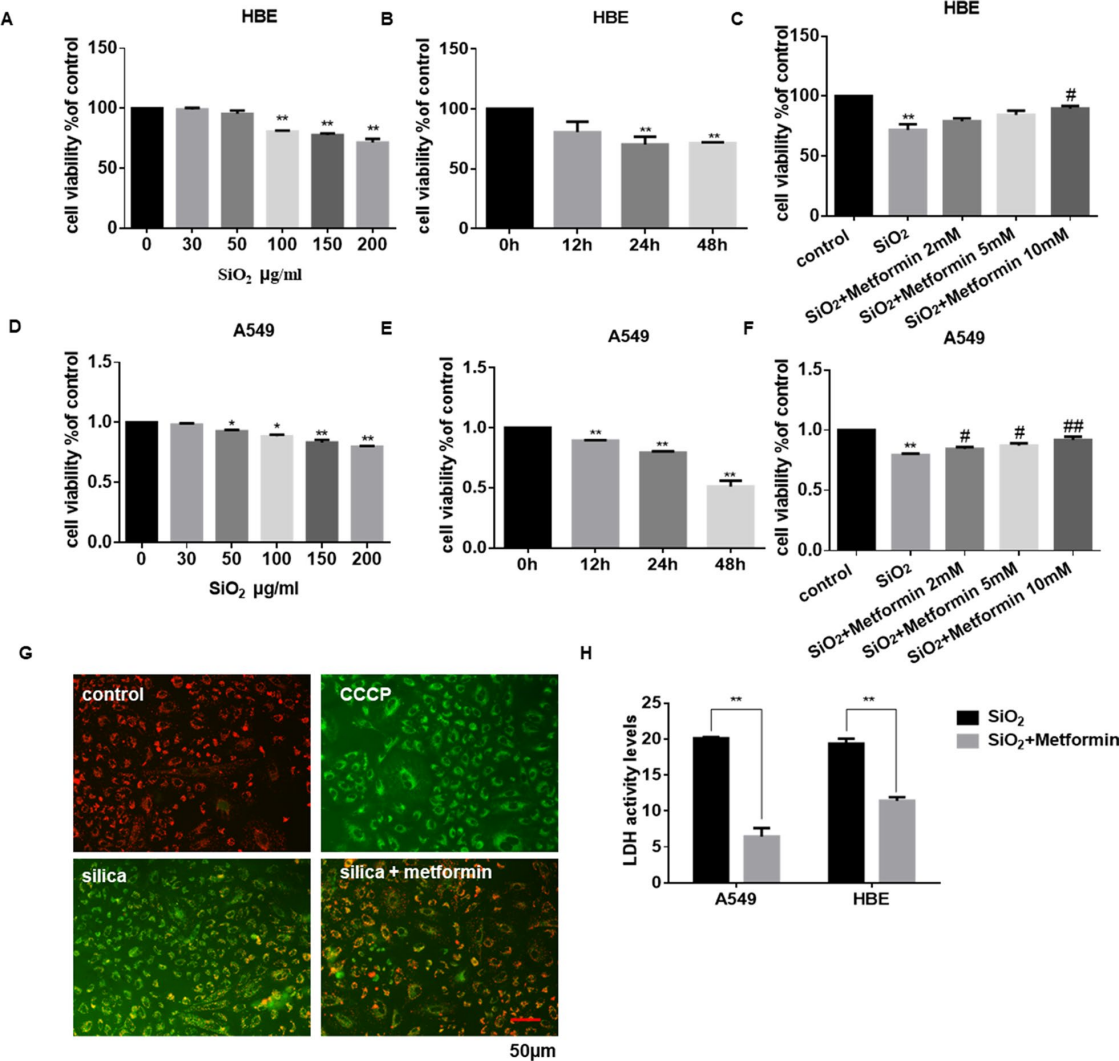
Metformin attenuates SiO_2_-induced cell cytotoxicity. **A**, **B**, **D** and **E** Cell viability was detected by cck8 assay in HBE and A549 cells. HBE (**A**) and A549 (**D**) cells were treated for 24 h in different concentrations of SiO_2_ (0–200 μg/ml). HBE (**B**) and A549 (**E**) cells were cultured with 200 μg/ml SiO_2_ for 12, 24, and 48 h separately. **C** and **F** HBE (**C**) and A549 (**F**) cells were incubated with 200 μg/ml SiO_2_ for 24 h with or without metformin (2–10 mM/ml). After that, the cell viability was analyzed by cck8 assay. **G** The mitochondrial membrane potential of A549 cells was measured by JC-1 staining. CCCP, the positive control; Green fluorescence, the monomer; red fluorescence, the J-aggregates; scale bar = 50 μm. **H** LDH activity of the A549 and HBE cells was measured using LDH assay, with **p < 0.01 vs. silica group

To further investigate the potential role of metformin in vitro, the cell cytotoxicity assay was performed to explore the optimal dose and time of silica and metformin. As shown in Fig. 3A, D, and Additional file 4: Figure S4A, the cell viability of HBE, A549, and THP-1 were gradually suppressed under the treatment of different concentrations of silica. Besides, the cytotoxicity of cells also has a time-dependent occurrence (Fig. 3B and E). The viability results suggested that the 200 μg/ml SiO_2_ 24 h treatment (cell viability decreased about 50%) showed moderate efficiency in HBE and A549 cells. This time dose was used in the rest of the experiments. As expected, co-treatment with various doses of metformin could inhibit HBE, A549 and THP-1 cells’ cytotoxicity induced by SiO_2_ (Fig. 3C and F; Additional file 4: Figure S4B).

Increased apoptosis was accompanied by the induction of SiO_2_ [35]. JC-1 staining showed that SiO_2_ induced epithelium cell apoptosis by depolarizing the mitochondrial membrane potential. However, co-treatment with metformin significantly reversed the disruption of mitochondrial membrane potential (Fig. 3G and Additional file 4: Figure S4C). Next, we used LDH release assay to investigate the viability of cells. Incubation of cells with SiO_2_ resulted in an apparent increase in LDH release; conversely, the presence of metformin significantly reduced LDH leakage (Fig. 3H). Together, these results suggested metformin protected cells from SiO_2_-induced cytotoxicity.

### Metformin inhibits SiO_2_-induced oxidative stress in vitro

**Fig. 4:**
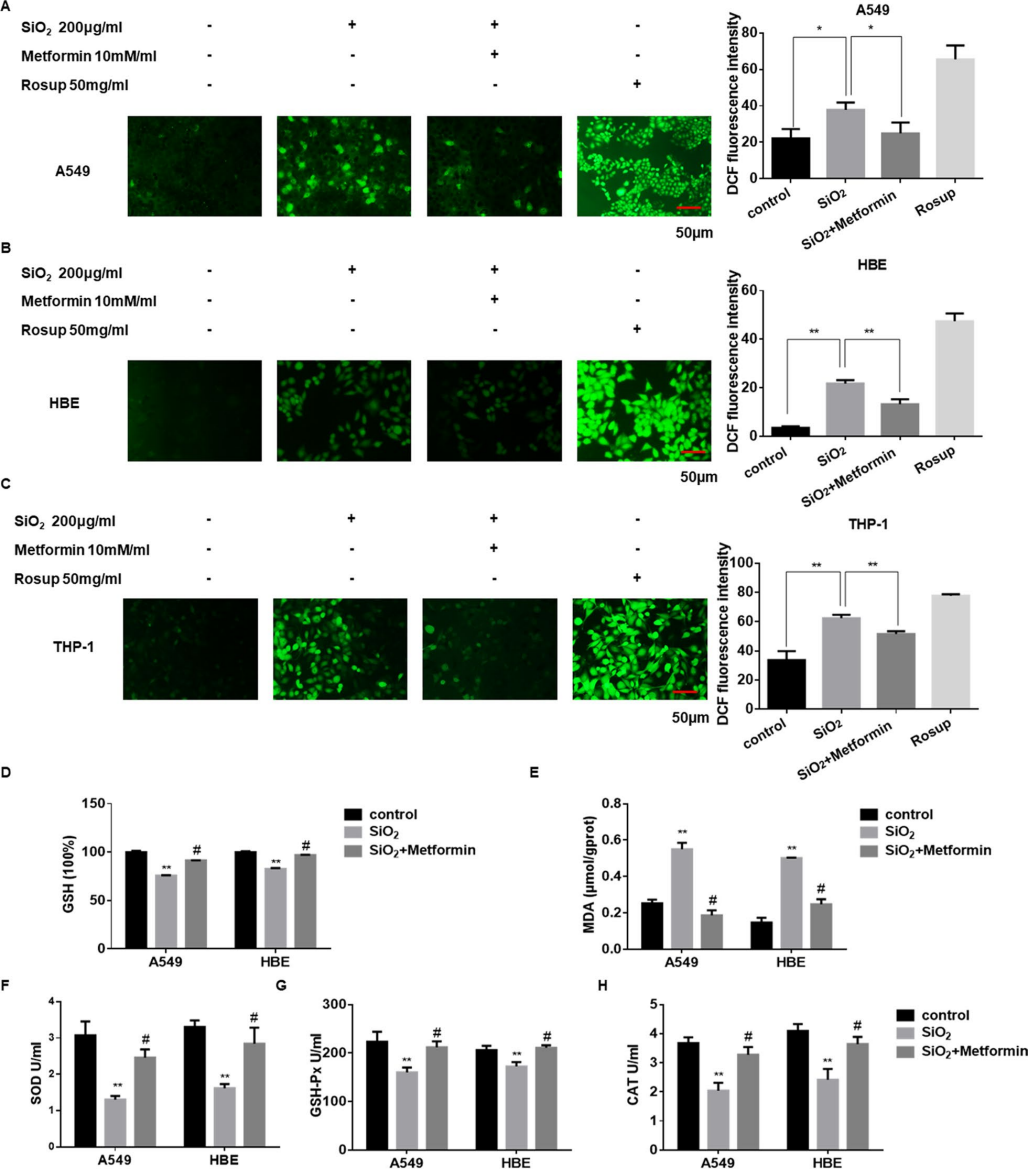
Metformin inhibits SiO_2_-induced oxidative stress. **A**–**C** The intracellular ROS generation of A549 (**A**), HBE (**B**), and THP-1 (**C**) cells were determined by the DCFH-DA probe, respectively. With *p < 0.05 and **p < 0.01 vs. control or silica group. **D**, **E** A549 and HBE cells were treated with silica and metformin for 24 h. The levels of GSH (**D**) and MDA (**E**) were determined by commercial kits. With **p < 0.01 vs. control and ^#^*p* < 0.05 vs. the silica group. The levels of SOD (**F**), GSH-Px (**G**) and CAT (**H**) in cell supernatant. With **p < 0.01 vs. control and ^#^*p* < 0.05 vs. the silica group

The generation of ROS is an essential indicator of oxidative stress and plays an important role in pulmonary fibrosis [36]. To examine the ROS generation in SiO_2_-treated cells, we used a DCFH-DA probe to detect cellular ROS production. As shown in Fig. 4A–C, the ROS generation was elevated after treatment with SiO_2_ (A549 and HBE cells for 24 h; THP-1 cells for 12 h) compared to controls, and this elevation was inhibited by co-treatment with metformin.

Glutathione (GSH) is one of the major antioxidant guardians in cellular, which could exert a critical role in reducing oxidative stress levels [37]. Recent reports have suggested that AMPK could induce peroxisome proliferator-activated receptor-gamma coactivator-1α (PGC-1α)-dependent glutamate-cysteine ligase modulatory subunit (GCLM) transcription to regulate GSH production [38]. To further explore the antioxidant ability of metformin, the oxidative damage in cells induced by SiO_2_ was measured. The antioxidant activities of GSH were decreased (Fig. 4D), whereas the lipid peroxidation levels of MDA were increased after SiO_2_ treatment (Fig. 4E). In contrast, treatment of metformin significantly enhanced GSH and decreased MDA content compared with the SiO_2_ treatment group. Besides, we also detected the levels of antioxidant enzymes like SOD, GSH-Px and CAT in cell supernatant. The levels of SOD, GSH-Px and CAT were increased after treatment of metformin compared with SiO_2_ treatment (Fig. 4F–H). Therefore, it appears that metformin may inhibit SiO_2_-induced oxidative stress in vitro.

### Metformin inhibits SiO_2_-induced pulmonary macrophage inflammatory response in vitro

**Fig. 5:**
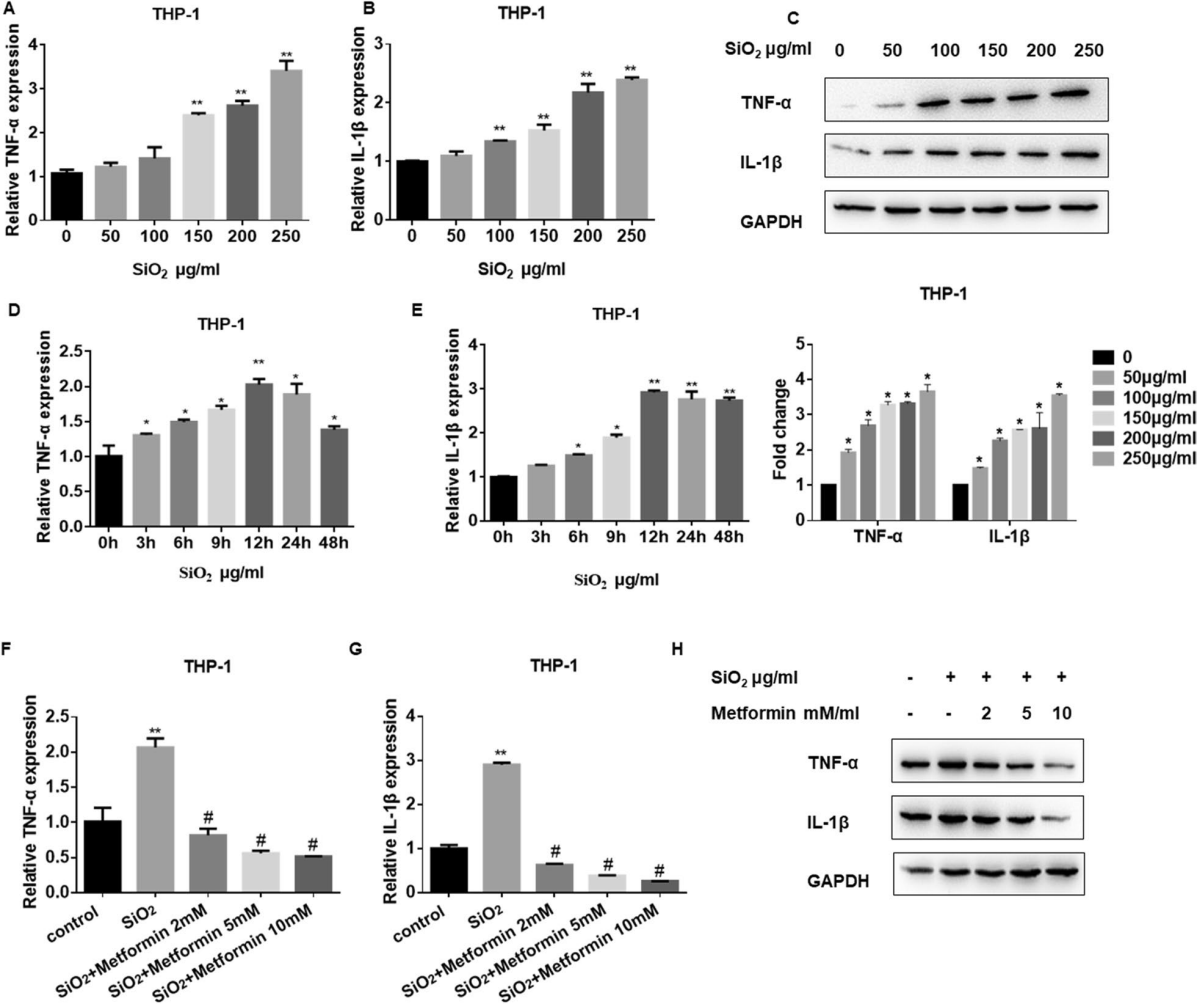
Metformin inhibits SiO_2_-induced pulmonary macrophage inflammatory response. **A**, **B**, **D** and **E** qRT-PCR detection of TNF-α (**A**, **D**) and IL-1β (**B**, **E**) mRNA expression in THP-1 cells from different concentration SiO_2_ and different time treatment, with *p < 0.05 and ^**^p < 0.01 vs. control group. **C** Western blots of TNF-α and IL-1β in THP-1 cells after incubated with SiO_2_. **F**, **G** qRT-PCR detection of TNF-α (**F**) and IL-1β (**G**) mRNA expression in THP-1 cells after silica and metformin co-treatment for 12 h, with **p < 0.01 vs. control and ^#^*p* < 0.05 vs. the silica group. **H** Western blots of TNF-α and IL-1β in THP-1 cells after incubated with SiO_2_ and metformin for 12 h

It has been shown that macrophages, and inflammatory mediators, including IL-1β and TNF-α, could contribute to exacerbating lung fibrosis [39]. Besides, previous studies have suggested that AMPK could attenuate lipopolysaccharide (LPS)-stimulated pro-inflammatory factors and increase anti-inflammatory cytokine levels by reducing the transcription activity of nuclear factor-kappaB (NF-κB) in macrophages [40]. Moreover, NF-κB could also exert a pro-inflammatory role in macrophages and result in the induction of inflammatory genes like IL-1β and TNF-α [41]. Thus, we detected the levels of inflammatory mediators in silica-stimulated macrophages by qRT-PCR analysis. The results showed that the mRNA levels of IL-1β and TNF-α were upregulated in a dose- (Fig. 5A and B) and time- (Fig. 5D and E) dependent manner. Besides, the western blot assay showed that the protein levels of IL-1β and TNF-α were also increased in SiO_2_-treated macrophages (Fig. 5C). Following metformin co-treatment, the mRNA (Fig. 5F and G) and protein (Fig. 5H and Additional file 5: Figure S5A) levels of IL-1β and TNF-α were significantly decreased compared with the SiO_2_-stimulated group. All the data indicate that metformin could be an effective therapy for SiO_2_-induced pulmonary macrophage inflammation.

### Metformin suppresses the SiO_2_-induced EMT process in lung epithelial cells in vitro

**Fig. 6:**
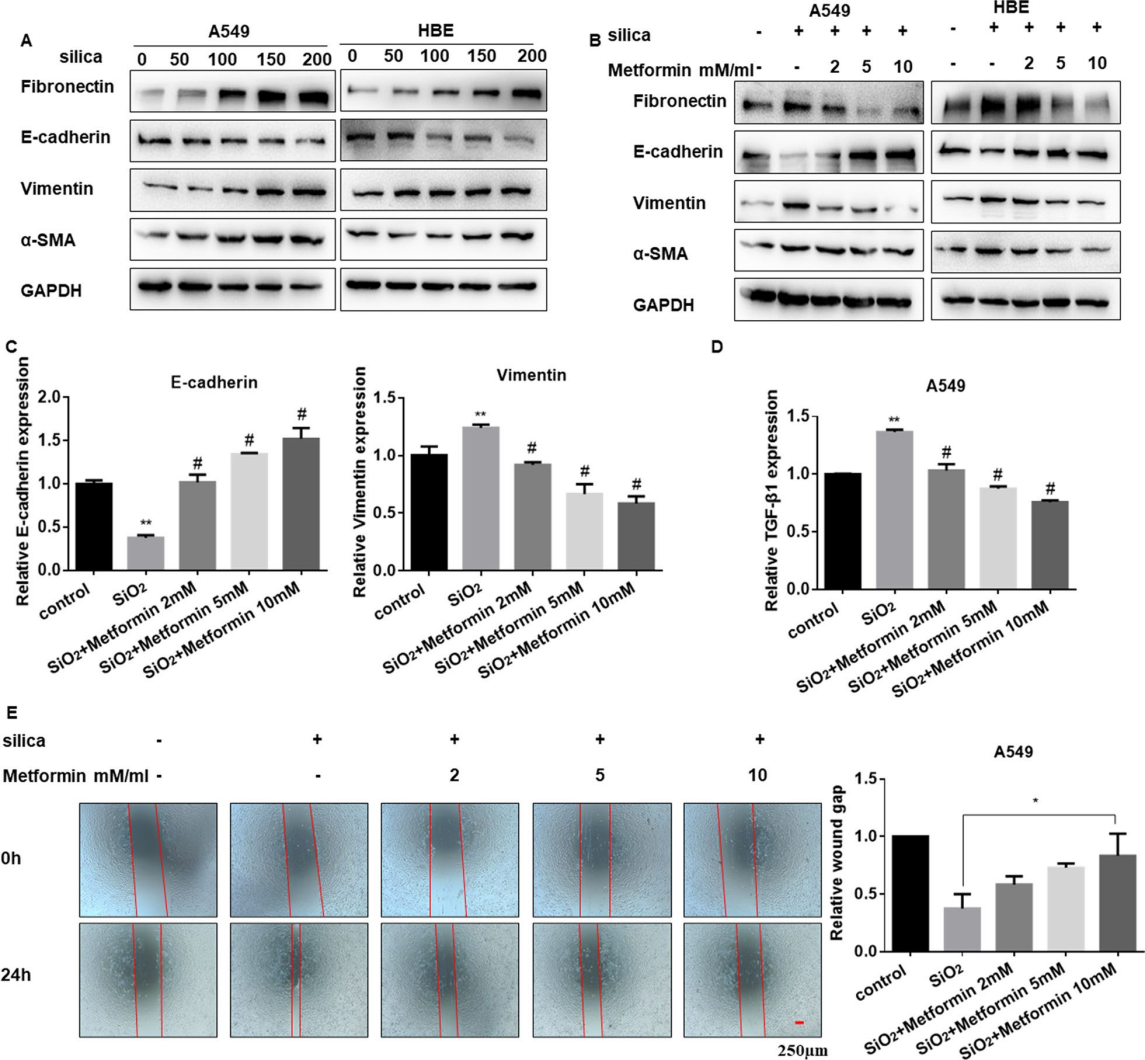
Metformin suppresses the SiO_2_-induced EMT process in lung epithelial cells. **A** The protein levels of Fibronectin, E-cadherin, vimentin and α-SMA in different doses of silica treatment in A549 and HBE cells. **B** Western blotting showing Fibronectin, E-cadherin, vimentin and α-SMA protein levels with silica and metformin co-treatment in A549 and HBE cells. **C**, **D** qRT-PCR detection of E-cadherin, vimentin (**C**) and TGF-β1 (**D**) mRNA expression in A549 cells after being cultured with silica and different doses of metformin. With **p < 0.01 vs. control and ^#^*p* < 0.05 vs. the silica group. **E** Wound-healing assay and quantified wound gap of cell scratches detected the migration of A549 cells for the indicated groups, with *p < 0.05 vs. the silica group

Next, we explored the appropriate dosage for SiO_2_ stimulation in A549 and HBE cells. Western blot results showed that the expression of α-SMA and vimentin were increased, while E-cadherin expression decreased in a dose-dependent manner (Fig. 6A and Additional file 6: Figure S6A, B), which indicated that the cell went through the EMT process. Meanwhile, metformin could enhance E-cadherin and down-regulated vimentin at protein (Fig. 6B and Additional file 6: Figure S6C, D) and mRNA levels (Fig. 6C). TGF-β1 is one of the main inducers of EMT [42, 43]. Our former research also reported that TGF-β1 could promote the EMT process in lung fibrosis [44]. In this study, we found that metformin also decreased the expression of TGF-β1 induced by SiO_2_ (Fig. 6D and Additional file 6: Figure S6E). Subsequently, the wound-healing assay was carried out to detect the migration ability of cells, which showed that the migration ability of A549 and HBE cells was increased in the presence of SiO_2_, while decreased in the intervention of metformin with a dose-dependent way (Fig. 6E and Additional file 6: Figure S6F). These data suggested that metformin could suppress the process of EMT in epithelial cells induced by SiO_2_.

### Metformin inhibits the TGF-β1-induced FMT process in pulmonary fibroblasts in vitro

Fibroblast-myofibroblast transition (FMT) is an essential pathological feature during pulmonary fibrosis, and FMT-derived myofibroblasts are the primary source of ECM components [45, 46]. Importantly, TGF-β1-induced FMT is the primary mechanism of fibrotic diseases.

**Fig. 7:**
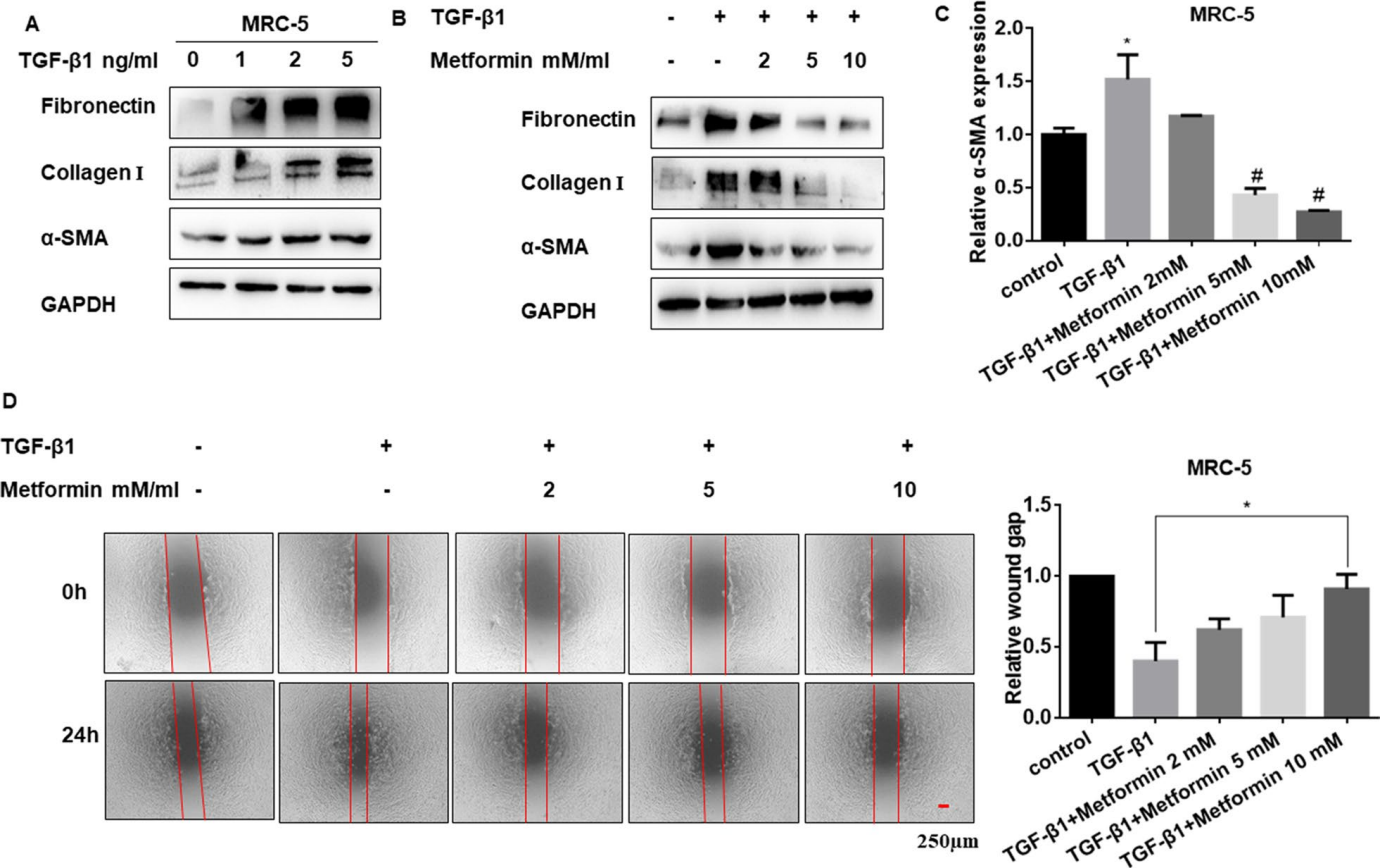
Metformin inhibits the TGF-β1-stimulated FMT process in pulmonary fibroblasts. **A** The protein levels of Fibronectin, Collagen I and α-SMA in MRC-5 cells are treated by different doses of TGF-β1. **B** Western blotting showing Fibronectin, Collagen I and α-SMA protein levels with TGF-β1 and metformin co-treatment in MRC-5 cells. **C** qRT-PCR detection of α-SMA mRNA expression in MRC-5 cells after cultured with TGF-β1 and different doses of metformin, with **p < 0.01 vs. control and ^#^*p* < 0.05 vs. the TGF-β1 group. **D** Wound-healing assay and quantified wound gap of cell scratches detected the migration of MRC-5 cells for the indicated groups, with *p < 0.05 vs. the TGF-β1 group

To explore the role of metformin on TGF-β1-stimulated FMT responses, we cultured MRC-5 cells with the presence of TGF-β1 to establish a cell FMT model. The western blot results showed that TGF-β1 dose-dependently increased the levels of fibrotic markers including Fibronectin, Collagen I and α-SMA (Fig. 7A and Additional file 7: Figure S7A). And it was also found that metformin could reverse the expression of fibrotic markers upregulated by TGF-β1 (Fig. 7B, C and Additional file 7: Figure S7B). The wound-healing assay showed that the migration of fibroblast cells induced by TGF-β1 was reversed by metformin (Fig. 7D). Overall, our results demonstrated that metformin could modulate the FMT process stimulated by TGF-β1 in fibroblast cells.

### Metformin protects against SiO_2_-induced lung fibrosis dependent on the AMPK pathway

**Fig. 8:**
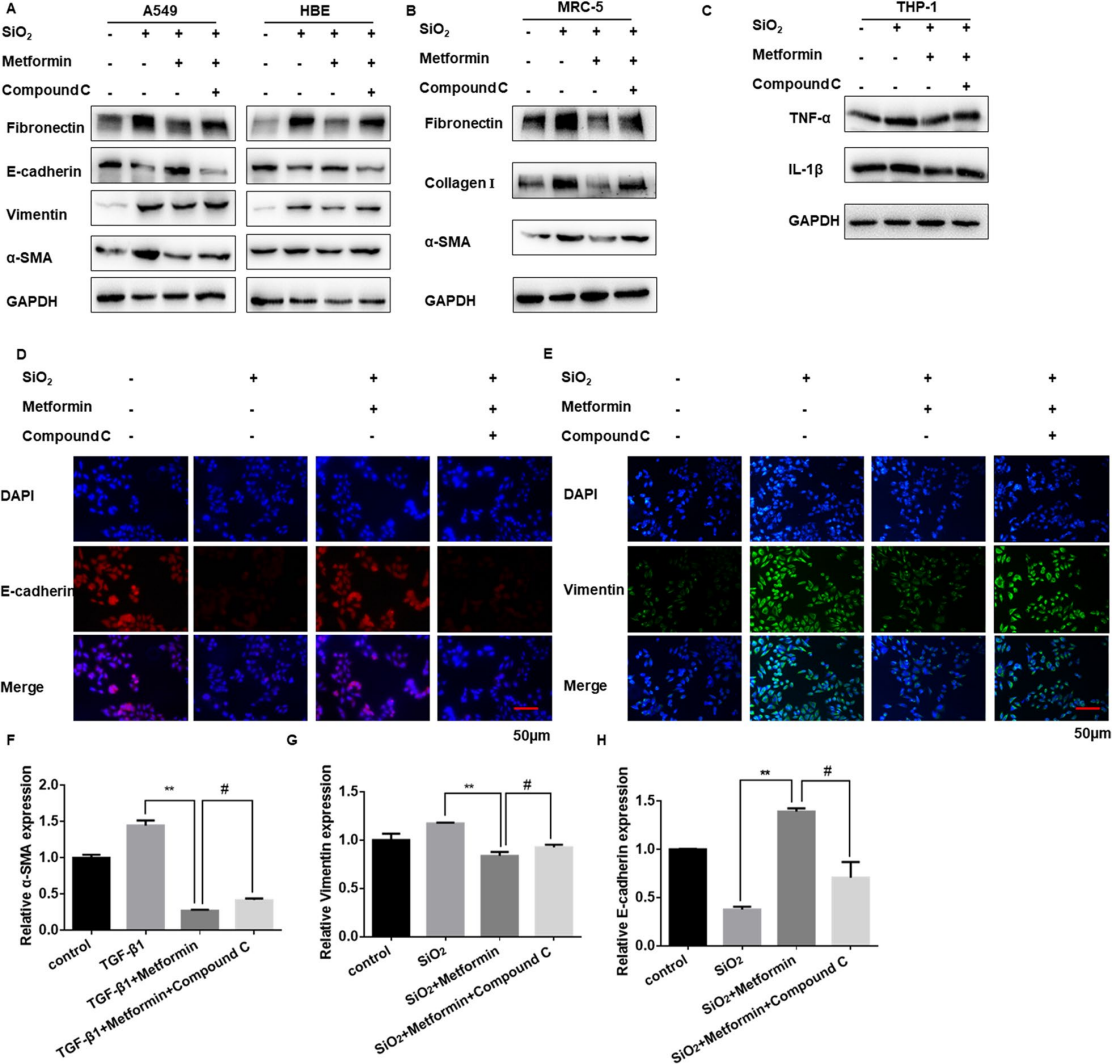
Metformin protects against SiO_2_-induced lung fibrosis dependent on the AMPK pathway. **A** The protein levels of Fibronectin, E-cadherin, vimentin, and α-SMA in A549 and HBE cells treated by silica, silica plus metformin and silica, metformin plus compound C. **B** Western blotting showing Fibronectin, Collagen I and α-SMA protein levels with different treatment in MRC-5 cell. **C** The protein levels of TNF-α and IL-1β in THP-1 cells for indicated groups. **D**, **E** Immunofluorescence staining of E-cadherin (**D**) and vimentin (**E**) in HBE cells in different groups. E-cadherin stained red, vimentin stained green, DAPI was stained blue, scale bar = 50 μm. **F**–**H** qRT-PCR detection of α-SMA (**F**), vimentin (**G**) and E-cadherin (**H**) mRNA expression for the indicated groups, with **p < 0.01 vs. TGF-β1 or silica group and ^#^*p* < 0.05 vs. the TGF-β1 or silica plus metformin group

To determine whether metformin plays its protective role in an AMPK-dependent manner, cells were treated with AMPK inhibitor Compound C. The effects of metformin in the process of EMT, FMT, and inflammation responses were reversed by Compound C (Fig. 8A–C and Additional file 8: Figure S8A, B). After metformin treatment, co-treatment with Compound C could reverse the EMT-blockage role of metformin (Fig. 8D, E and Additional file 8: Figure S8C). Moreover, treatment with metformin significantly reduced TGF-β1-induced the expression of α-SMA in MRC-5 cells, which was also reversed by Compound C (Fig. 8F and Additional file 8: Figure S8D). Similarly, after metformin treatment, the mRNA expression of vimentin, as well as E-cadherin, was also reversed by Compound C (Fig. 8G, H).

Based on other studies, we found that AMPK could regulate macrophages differentiation, EMT process, myofibroblasts activation via inhibiting signal transducer and activator of transcription 3 (STAT3) [47–49]. Therefore, we used AMPK siRNA to detected the level of TGF-β1, STAT3 and downstream molecules in our cell models. Our data showed that the high expression of phosphorylated signal transducer and activator of transcription 3 (p-STAT3), TGF-β1, inflammatory cytokines, fibrotic markers and EMT-associated molecules (E-cadherin was decreased) in AMPK siRNA and TGF-β1 or silica-treated group (Additional file 8: Figure S8E-G). Taken together, we speculated that metformin inhibits pulmonary fibrosis by AMPK-regulated STAT3 activation.

Taken together, these data indicated that metformin affects SiO_2_-induced pulmonary fibrosis in an AMPK dependent manner.

## Discussion

Silicosis is one of the potentially fatal lung diseases, mainly caused by long-term inhalation of respirable crystalline free silica (SiO_2_) in occupational environments [50]. Recent findings exist that the progression of silicosis is a complex process. For example, Yi Zhang et al. have found that an imbalance between N‑acetyl‑seryl‑aspartyl‑lysyl‑proline (Ac-SDKP) and angiotensin II (Ang II) could promote the development of silicosis [51]. The essential characters of silicosis include the chronic inflammation response, and the continuous aggravation of the fibrotic process, eventually leading to respiratory failure [52]. Although attempts to prevent silicosis have been made for several decades, it remains a severe public health problem worldwide, especially in developing countries [53]. Currently, effective clinical therapies are limited. Few drugs can reverse the progression of silicosis. Even though lung transplantation is an effective intervention shown to provide the longest survival time, it is associated with the low availability of suitable lung donors and disease-specific challenges [54]. It is urgent to identify useful drugs to attenuate the development of silicosis. Hence, in this study, we established metformin intervention models in vitro and in vivo to explore its potential anti-fibrotic effects.

Metformin, a widely used miracle drug, is well known for treating type 2 diabetes. However, with advances in epidemiological and experimental researches, growing evidence suggests that metformin has multiple benefits in various diseases, including fibrotic diseases [55–57]. For example, metformin was found to alter the fate of myofibroblasts by inducing lipogenic differentiation and inhibit TGF-β1-stimulated myogenic differentiation [58]. Besides, recent studies have shown that metformin could prevent age-associated ovarian fibrosis and exert anti-ovarian cancer effects [59]. Moreover, abundant data have indicated that metformin might attenuate PM_2.5_-induced lung injury and cardiac fibrosis [60]. Thus, these findings supported that metformin acted as a potential anti-fibrotic drug. In the present study, the positive effect of metformin was further investigated in the silica-induced fibrosis mouse model. However, the metformin pharmacogenetic study suggests that metformin is mainly concentrated in the gut even after absorption and is unlikely to reach the lung tissues with high concentrations using the oral supplementation route [29]. To better enrich the drug in the lungs and decrease the usage dosage, metformin inhalation therapy may achieve better results in the future with fewer side effects.

The inhalation of respiratory silica dust can induce inflammatory responses, the secretion of inflammatory factors, and high ROS levels, which contribute to chronic lung injury [35]. It has been reported that metformin could inhibit the production of pro-inflammatory cytokines such as TNF-α from macrophages by inhibiting NF-κB [61]. Moreover, the overproduction of intracellular ROS promotes the activation of alveolar macrophages and the secretion of pro-inflammatory cytokines, which aggravates lung dysfunction [62]. In this study, we found that SiO_2_ exposure significantly increased the level of intracellular ROS and the release of pro-inflammatory cytokines, which was effectively attenuated after the metformin treatment. Similarly, other studies have also elucidated the antioxidative and anti-inflammatory properties of metformin in different experimental models [63–65]. For example, metformin attenuates hypoxia-induced mitochondrial ROS in different cell lines by modulating the hypoxia-inducible factor-1alpha (HIF-1α) pathway [66]. Additionally, the finding that metformin reversed SiO_2_-induced levels of GSH, SOD, CAT, GSH-Px, MDA and inflammatory cytokines suggested that metformin could acted as a useful antioxidant and anti-inflammatory agent.

Numerous studies have demonstrated that the contribution of EMT in fibrosis diseases, including pulmonary fibrosis [67]. Usually, EMT was one of the major sources of fibroblasts in various tissues. To evaluate the potential role of EMT in silicosis, our previous studies established a co-culture system by culturing SiO_2_ stimulated-epithelial cells with fibroblasts [68]. In the present study, we found that metformin upregulated E-cadherin level and downregulated the level of vimentin induced by SiO_2_. Therefore, these results indicated that metformin acts as an effective medicine by halting the progression of EMT. Notably, the inhibitor of metformin, Compound C, could reverse the metformin-blocked EMT process. Also, metformin is involved in a variety of diseases by regulating the process of EMT. For example, metformin decreases cell invasion, growth, and EMT via modulation of miR-381/Yes-associated protein (YAP) activity in non-small cell lung cancer [69]. Metformin also suppressed dipeptidyl peptidase-4 (DPP-4) inhibitor-induced EMT via the suppression of mechanistic target of rapamycin (mTOR) signaling in breast cancer [70].

The aberrant activation of fibroblast associated with excessive extracellular matrix accumulation is the main characteristic of pulmonary fibrosis [71]. As the master regulator of fibrosis, TGF-β1 could directly induce transcription of profibrotic molecules including α-SMA, fibronectin, and collagens via TGF-β1/Smad signaling during myofibroblast activation [72]. Also, TGF-β1 acts as the promoter of myofibroblast differentiation and regulates the expression of α-SMA, fibronectin, and collagen type I [73]. In this study, we found that the treatment of metformin significantly inhibited TGF-β1-induced myofibroblast differentiation. And, metformin regulated TGF-β1-induced fibrotic markers expression at protein and mRNA level, which was reversed by Compound C. It has been reported that activation of AMPK via metformin could reduce TGF-β1-stimulated collagen type I by inhibiting Smad3 signaling [74]. Meanwhile, Jiamei Lu et al. [75] presented that baicalin could attenuate cardiac fibrosis by mediating the AMPK inhibited TGF-β1/Smads signaling. Sachin Thakur et al. [76] showed that AMPK activation by metformin could decrease TGF-β1-induced α-SMA and fibronectin expression. Hence, we speculated that metformin-mediated AMPK activation could inhibit the process of TGF-β1-induced myofibroblast differentiation. Besides, it has been reported that metformin could reduce TGF-β1-treated ECM protein accumulation in nasal polys-derived fibroblasts [77].

However, it is widely recognized that the inhibitor of AMPK, Compound C, has other actions. Previous researches indicated that Compound C also acted as a small-molecule inhibitor of BMP signaling, and blocked bone morphogenetic protein (BMP)-mediated target gene transcription and SMAD1/5/8 phosphorylation [78]. While, TGF-β and BMP are belonging to the TGF-β superfamily, and the reduction of BMP might cause the increased expression of TGF-β and drive the process of fibrosis [79, 80]. Therefore, we further used AMPK siRNA to explore the role of AMPK in lung fibrosis.

Growing evidence has identified that metformin exerts its pathology and physiology functions via both AMPK-dependent and AMPK-independent pathways. However, we only researched metformin to attenuate silica-induced lung fibrosis through AMPK-dependent inhibition of STAT3 expression. However, STAT3 might be only one of many AMPK downstream signals. AMPK is heavily involved in energy homeostasis, autophagy, cellular stress resistance, inflammatory response and fibrosis through its regulation of multiple downstream targets, like mTOR, (Forkhead box O) FOXO, NF-κB and TGF-β1 signaling [81, 82]. Further study is needed to focus on the other potential downstream effectors of the AMPK pathways in lung fibrosis.

## Conclusions

**Fig. 9:**
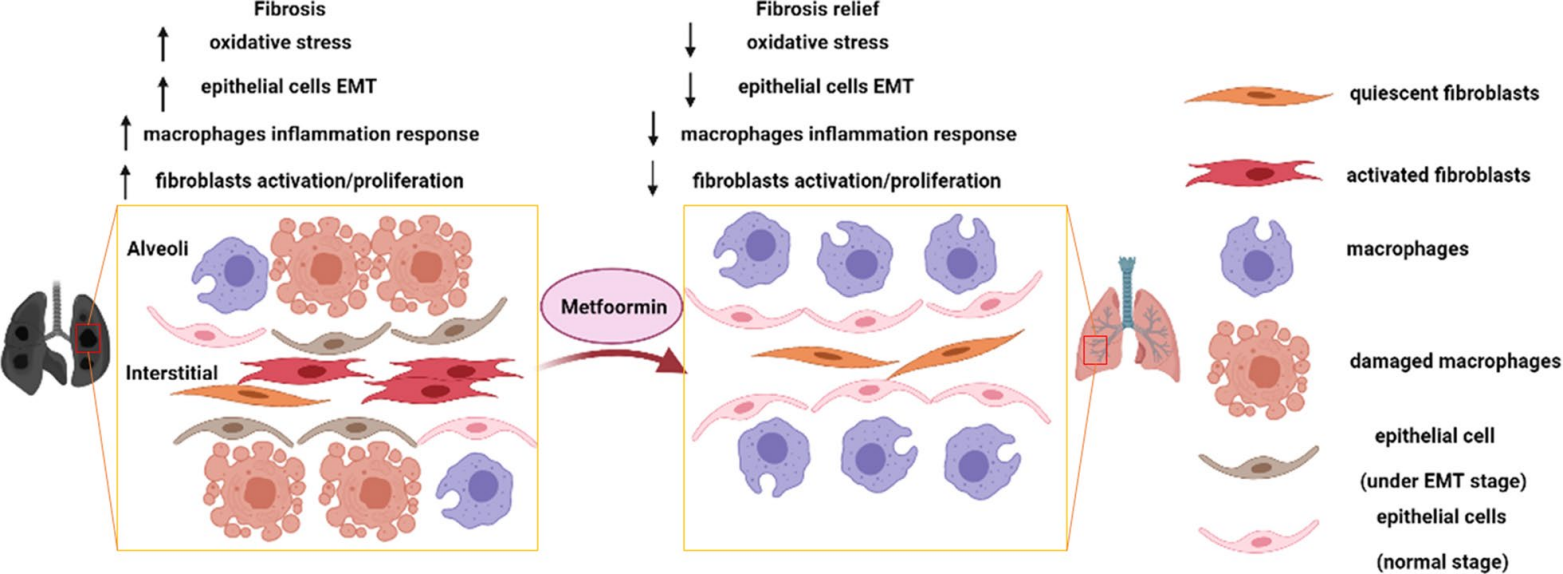
Schematic illustration represented the role of metformin in pulmonary fibrosis. Created with https://BioRender.com

Our finding indicated that metformin, dependent on AMPK signaling, attenuated silica-induced pulmonary fibrosis by decreasing cell toxicity, inflammation, oxidative stress, EMT, and fibroblast activation processes (Fig. 9). These results suggest that metformin administration is a potential therapy in fibrotic relative diseases.

## Supplementary Information

**Additional file 1: Figure S1.** Metformin/AMPK related signalings in fibrotic diseases.

**Additional file 2: Figure S2.** Metformin attenuates SiO_2_-induced lung fibrosis in vivo. (A) The body weight of the mice in each group. (B, C) qRT-PCR detection of TNF-α (A) and IL-1β (B) mRNA expression in lung tissues for the indicated groups, with ^*^p < 0.05 vs. control and ^#^*p* < 0.05 vs. silica group.

**Additional file 3: Figure S3.** Metformin attenuates SiO_2_-induced lung fibrosis in vivo. (A) H&E staining reflected that the histological changes of lung tissues for the indicated groups. (B) Densitometric analysis of Fibronectin, Collagen I, E-cadherin, vimentin and α-SMA in lung tissues, with ^*^p < 0.05 vs. control and ^#^*p* < 0.05 vs. silica plus saline group. (C, D) qRT-PCR detection of TNF-αand IL-1β mRNA expression in lung tissues, with ^**^p < 0.01 vs. control and ^#^*p* < 0.05 vs. silica plus saline group.

**Additional file 4: Figure S4.** Metformin attenuates SiO_2_-induced cell cytotoxicity. (A, B) Cell viability was detected by cck8 assay in THP-1 cells for the indicated groups, with ^**^p < 0.01 vs. control and ^#^*p* < 0.05 vs. silica group. (C) The mitochondrial membrane potential of HBE cells was measured by JC-1 staining. CCCP: the positive control, Green fluorescence: the monomer, red fluorescence: the J-aggregates, scale bar = 50 μm.

**Additional file 5: Figure S5.** Metformin inhibits SiO_2_-induced pulmonary macrophage inflammatory response. (A) Densitometric analysis of TNF-α and IL-1β in THP-1 cells for the indicated groups, with ^**^p < 0.01 vs. control and ^#^*p* < 0.05 vs. silica group.

**Additional file 6: Figure S6.** Metformin suppresses the SiO_2_-induced EMT process in lung epithelial cells. (A-D) Densitometric analysis of Fibronectin, E-cadherin, vimentin, and α-SMA in A549 and HBE cells for the indicated groups, with ^*^p < 0.05 vs. control or silica group. (E) qRT-PCR analysis of TGF-β1 in HBE cells for different treatment, with ^**^p < 0.01 vs. control and ^#^*p* < 0.05 vs. silica group. (F) Quantified wound gap of wound healing to detect the migration of HBE cells for the indicated groups, with ^*^p < 0.05 vs. the silica group.

**Additional file 7: Figure S7.** Metformin inhibits the TGF-β1-stimulated FMT process in pulmonary fibroblasts. (A, B) Densitometric analysis of Fibronectin, Collagen I and α-SMA in MRC-5 cells for the different groups, with ^*^p < 0.05 vs. control and ^#^*p* < 0.05 vs. TGF-β1 group.

**Additional file 8: Figure S8.** Metformin protects against SiO_2_-induced lung fibrosis dependent on the AMPK pathway. (A, B) Densitometric analysis of Fibronectin, E-cadherin, vimentin, and α-SMA A549 and HBE cells for the indicated groups, with ^*^p < 0.05 vs. silica and ^#^*p* < 0.05 vs. silica plus metformin group. (C) Immunofluorescence staining of E-cadherin in A549 cells in different groups. E-cadherin stained red, DAPI was stained blue, scale bar = 50 μm. (D) Immunofluorescence staining of α-SMA in MRC-5 cells in different groups. α-SMA stained red, DAPI was stained blue, scale bar = 50 μm. (E–G) The protein levels of the indicated index in A549, HBE, MRC-5 and THP-1 cells.

## Abbreviations

AMPK: AMP-activated protein kinase
BMP: Bone morphogenetic protein
CAT: Catalase
DPP-4: Dipeptidyl peptidase-4
ECM: Extracellular matrix
EMT: Epithelial-mesenchymal transition
FMT: Fibroblast-to-myofibroblast transition
FOXO: Forkhead box O
GCLM: Glutamate-cysteine ligase modifier subunit
GSH: Glutathione
GSH-Px: Glutathione peroxidase
H&E: Hematoxylin and eosin
HIF-1α: Hypoxia-inducible factor-1alpha
IL-1β: Interleukin-1 beta
LDH: Lactate dehydrogenase
LPS: Lipopolysaccharide
MDA: Malondialdehyde
mTOR: Mechanistic target of rapamycin
NF-κB: Nuclear factor-kappaB
PGC-1α: Peroxisome proliferator-activated receptor-gamma coactivator-1 alpha
P-STAT3: Phosphorylated signal transducer and activator of transcription 3
ROS: Reactive oxygen species
SiO_2_: Silicon dioxide
SMAD: Small mothers against decapentaplegic
SOD: Superoxide dismutase
STAT3: Signal transducer and activator of transcription 3
T2DM: Type 2 diabetes mellitus
TGF-β1: Transforming growth factor-beta 1
TNF-α: Tumor necrosis factor-alpha
YAP: Yes-associated protein
