# Supplementary figures and images for: Metformin attenuates silica-induced pulmonary fibrosis via AMPK signaling
Source: J Transl Med. 2021 Aug 16;19:349. doi: 10.1186/s12967-021-03036-5 (PMC8365894; doi:10.1186/s12967-021-03036-5)

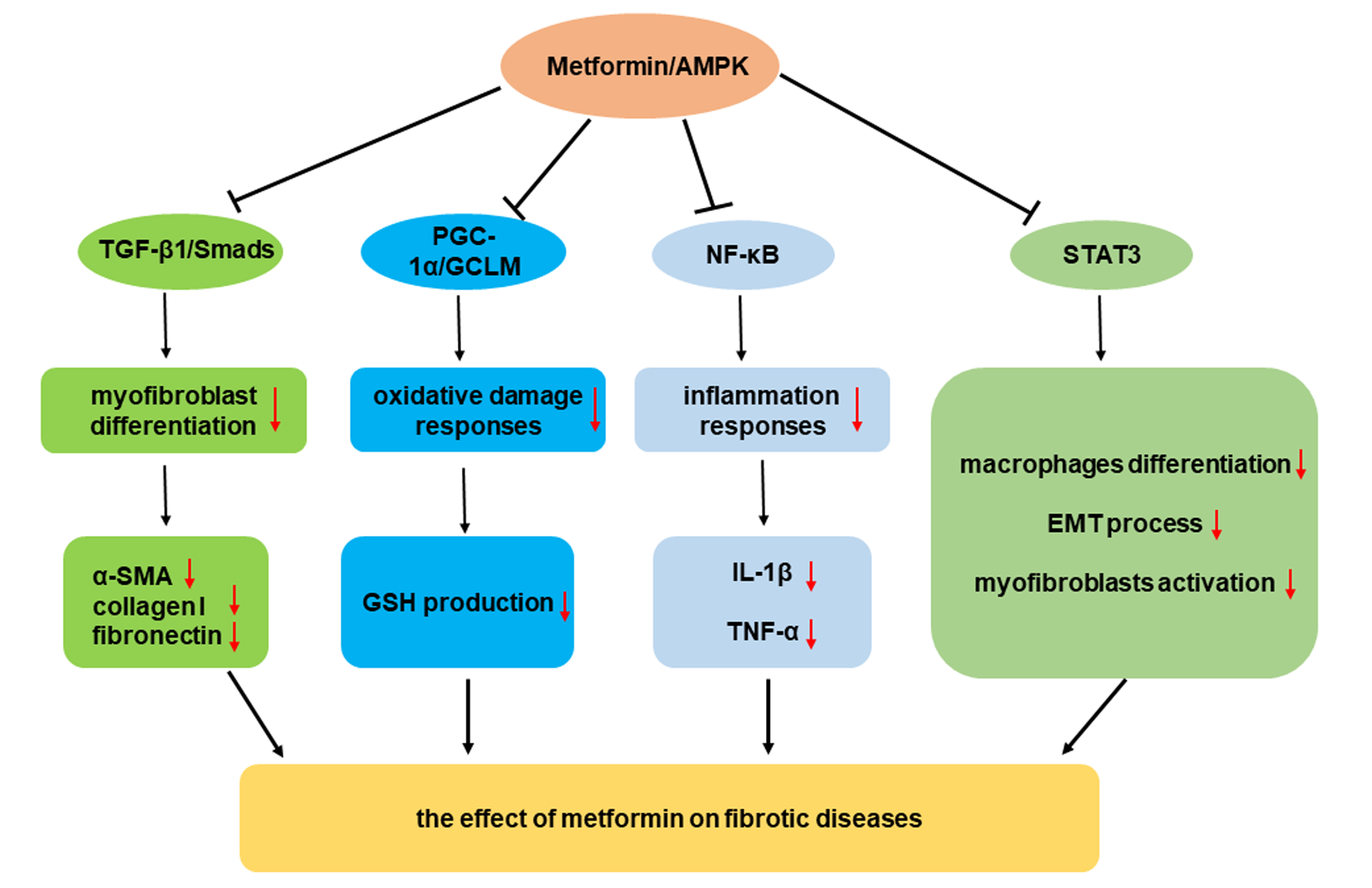

Supplement: Supplementary file 1 — Additional file 1: Figure S1. Metformin/AMPK related signalings in fibrotic diseases. [file 12967_2021_3036_MOESM1_ESM.tif]

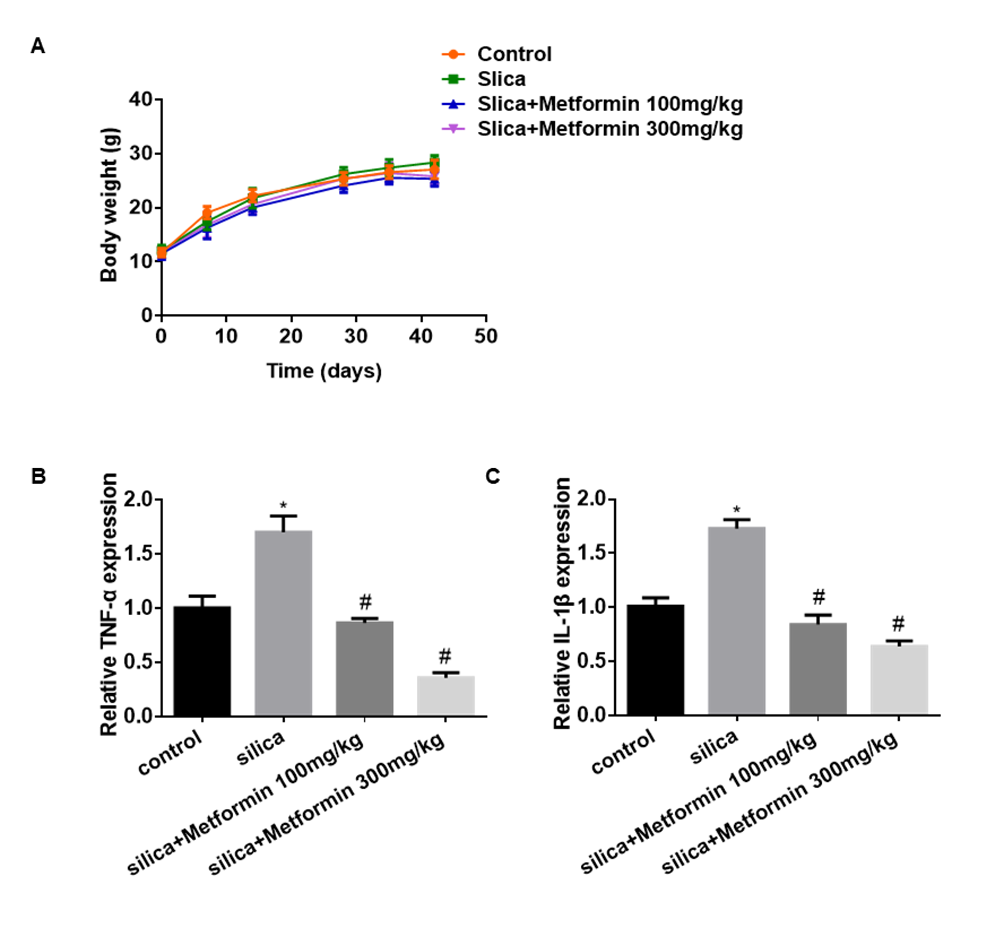

Supplement: Supplementary file 2 — Additional file 2: Figure S2. Metformin attenuates SiO2-induced lung fibrosis in vivo. (A) The body weight of the mice in each group. (B, C) qRT-PCR detection of TNF-α (A) and IL-1β (B) mRNA expression in lung tissues for the indicated groups, with *p < 0.05 vs. control and #p < 0.05 vs. silica group. [file 12967_2021_3036_MOESM2_ESM.tif]

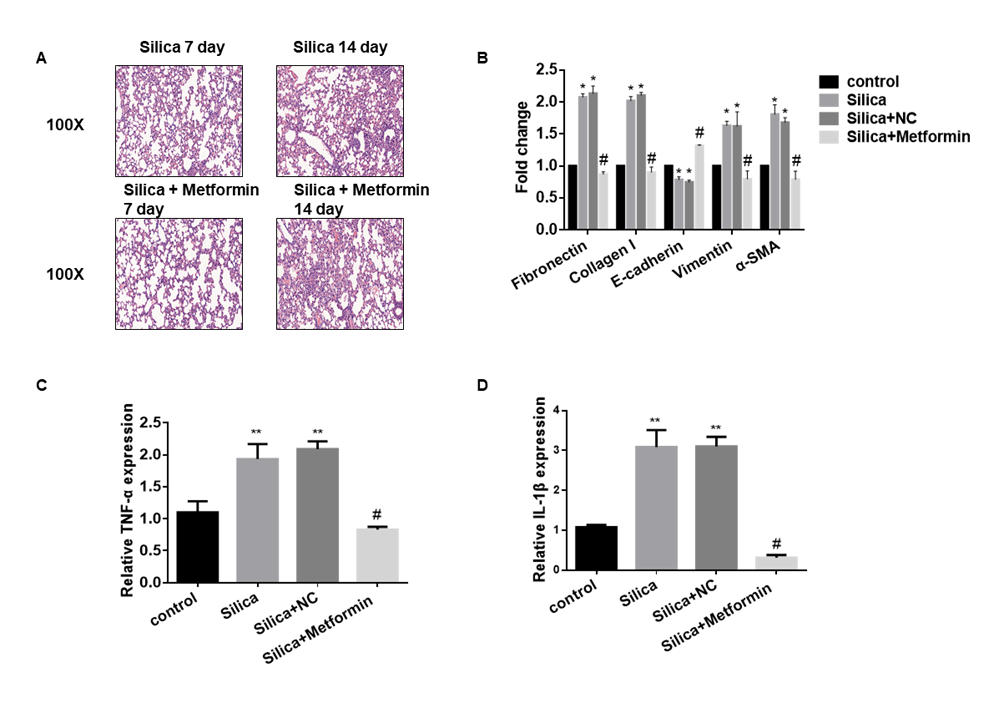

Supplement: Supplementary file 3 — Additional file 3: Figure S3. Metformin attenuates SiO2-induced lung fibrosis in vivo. (A) H&E staining reflected that the histological changes of lung tissues for the indicated groups. (B) Densitometric analysis of Fibronectin, Collagen I, E-cadherin, vimentin and α-SMA in lung tissues, with *p < 0.05 vs. control and #p < 0.05 vs. silica plus saline group. (C, D) qRT-PCR detection of TNF-αand IL-1β mRNA expression in lung tissues, with **p < 0.01 vs. control and #p < 0.05 vs. silica plus saline group. [file 12967_2021_3036_MOESM3_ESM.tif]

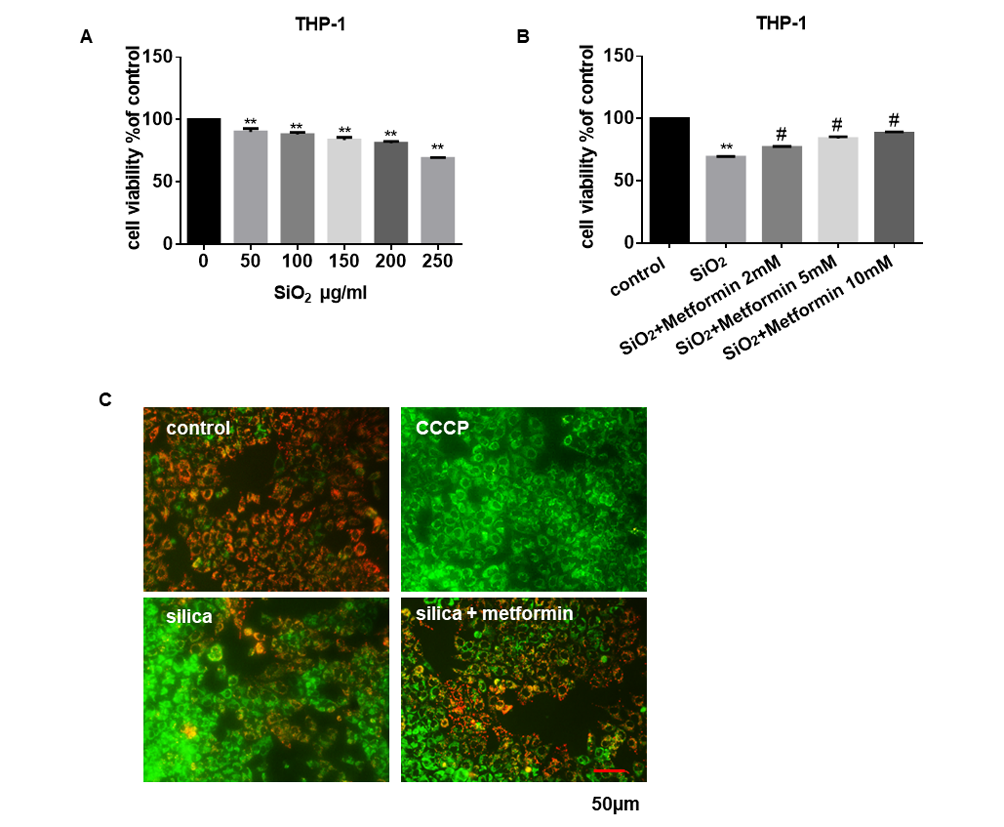

Supplement: Supplementary file 4 — Additional file 4: Figure S4. Metformin attenuates SiO2-induced cell cytotoxicity. (A, B) Cell viability was detected by cck8 assay in THP-1 cells for the indicated groups, with **p < 0.01 vs. control and #p < 0.05 vs. silica group. (C) The mitochondrial membrane potential of HBE cells was measured by JC-1 staining. CCCP: the positive control, Green fluorescence: the monomer, red fluorescence: the J-aggregates, scale bar = 50 μm. [file 12967_2021_3036_MOESM4_ESM.tif]

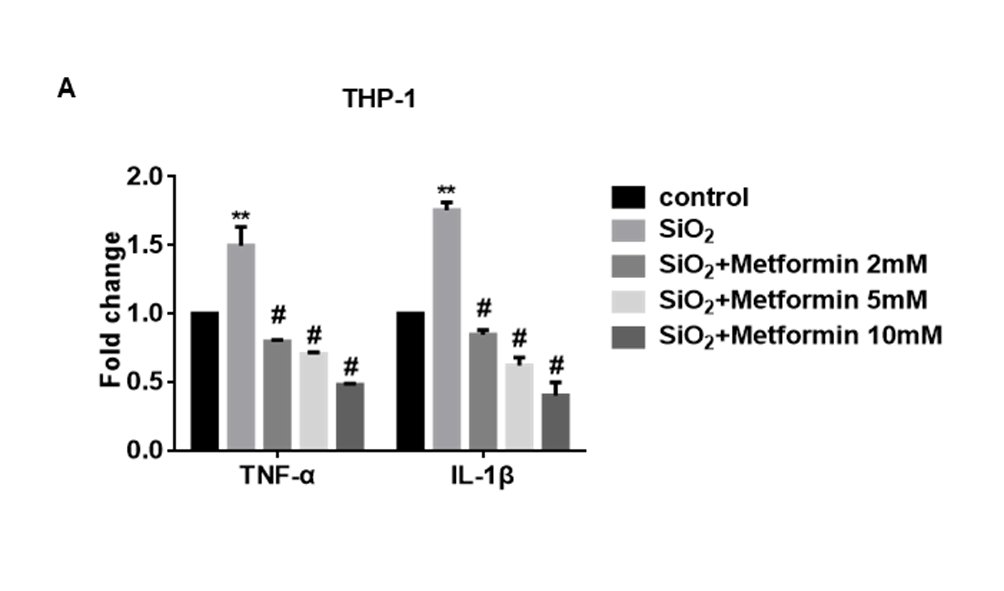

Supplement: Supplementary file 5 — Additional file 5: Figure S5. Metformin inhibits SiO2-induced pulmonary macrophage inflammatory response. (A) Densitometric analysis of TNF-α and IL-1β in THP-1 cells for the indicated groups, with **p < 0.01 vs. control and #p < 0.05 vs. silica group. [file 12967_2021_3036_MOESM5_ESM.tif]

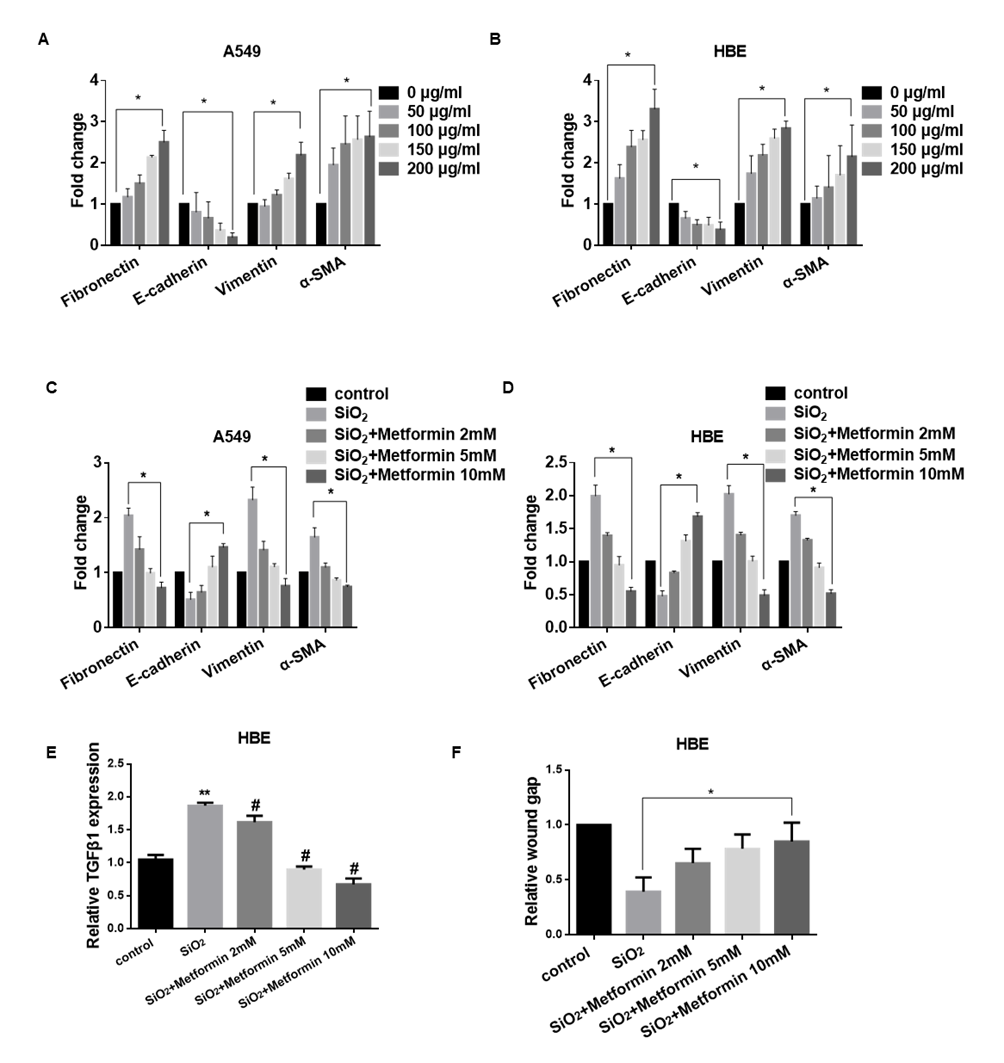

Supplement: Supplementary file 6 — Additional file 6: Figure S6. Metformin suppresses the SiO2-induced EMT process in lung epithelial cells. (A-D) Densitometric analysis of Fibronectin, E-cadherin, vimentin, and α-SMA in A549 and HBE cells for the indicated groups, with *p < 0.05 vs. control or silica group. (E) qRT-PCR analysis of TGF-β1 in HBE cells for different treatment, with **p < 0.01 vs. control and #p < 0.05 vs. silica group. (F) Quantified wound gap of wound healing to detect the migration of HBE cells for the indicated groups, with *p < 0.05 vs. the silica group. [file 12967_2021_3036_MOESM6_ESM.tif]

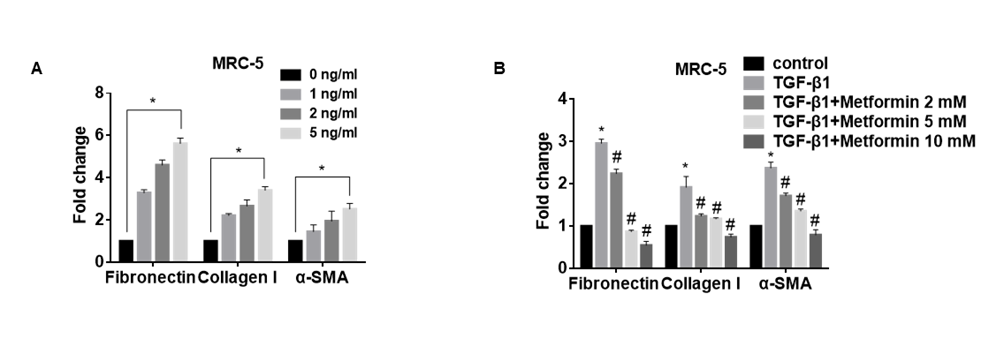

Supplement: Supplementary file 7 — Additional file 7: Figure S7. Metformin inhibits the TGF-β1-stimulated FMT process in pulmonary fibroblasts. (A, B) Densitometric analysis of Fibronectin, Collagen I and α-SMA in MRC-5 cells for the different groups, with *p < 0.05 vs. control and #p < 0.05 vs. TGF-β1 group. [file 12967_2021_3036_MOESM7_ESM.tif]

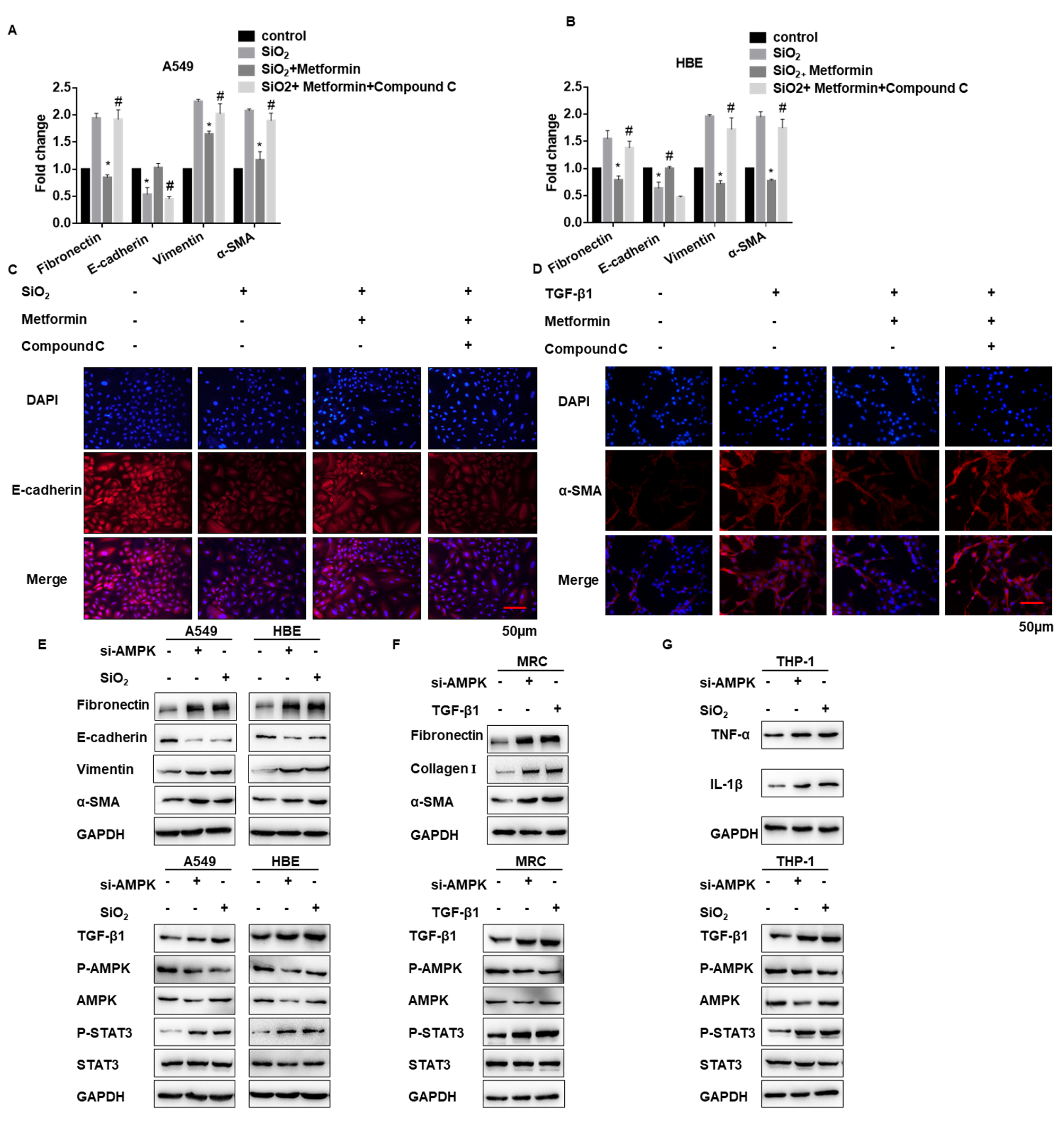

Supplement: Supplementary file 8 — Additional file 8: Figure S8. Metformin protects against SiO2-induced lung fibrosis dependent on the AMPK pathway. (A, B) Densitometric analysis of Fibronectin, E-cadherin, vimentin, and α-SMA A549 and HBE cells for the indicated groups, with *p < 0.05 vs. silica and #p < 0.05 vs. silica plus metformin group. (C) Immunofluorescence staining of E-cadherin in A549 cells in different groups. E-cadherin stained red, DAPI was stained blue, scale bar = 50 μm. (D) Immunofluorescence staining of α-SMA in MRC-5 cells in different groups. α-SMA stained red, DAPI was stained blue, scale bar = 50 μm. (E–G) The protein levels of the indicated index in A549, HBE, MRC-5 and THP-1 cells. [file 12967_2021_3036_MOESM8_ESM.tif]
